# Male proband with intractable seizures and a *de novo* start-codon-disrupting variant in *GLUL*

**DOI:** 10.1016/j.xhgg.2025.100419

**Published:** 2025-02-21

**Authors:** Elizabeth Carbonell, Sarah L. Stenton, Vijay S. Ganesh, Jialan Ma, Grace E. VanNoy, Lynn Pais, John N. Gaitanis, Melanie C. O’Leary, Heidi L. Rehm, Anne O’Donnell-Luria

**Affiliations:** 1 Program in Medical and Population Genetics, Broad Institute of MIT and Harvard, Cambridge, MA, USA; 2 Division of Genetics and Genomics, Boston Children’s Hospital, Harvard Medical School, Boston, MA, USA; 3 Department of Neurology, Brigham and Women’s Hospital, Harvard Medical School, Boston, MA, USA; 4 Hasbro Children’s Hospital, The Warren Alpert Medical School of Brown University, Providence, RI, USA; 5 Center for Genomic Medicine, Massachusetts General Hospital, Harvard Medical School, Boston, MA USA

**Keywords:** GLUL, glutamine synthetase, de novo, start-loss, start codon, 5'UTR, seizures

## Abstract

Bi-allelic variants in *GLUL*, encoding glutamine synthetase and responsible for the conversion of glutamate to glutamine, are associated with a severe recessive disease due to glutamine deficiency. A dominant disease mechanism was recently reported in nine females, all with a *de novo* single-nucleotide variant within the start codon or the 5′ UTR of *GLUL* that truncates 17 amino acids of the protein product, including its critical N-terminal degron sequence. This truncation results in a disorder of abnormal glutamine synthetase stability and manifests as a phenotype of severe developmental and epileptic encephalopathy. Here, we report the first male with a pathogenic *de novo* variant in the same critical region of *GLUL*, with a phenotype of refractory focal and generalized seizures, as well as developmental delays. We provide a detailed description of the disease course and treatment response.

## Teaser

*De novo* start-loss variants in the start codon or 5′ UTR of *GLUL* result in abnormal glutamine synthetase stability and have been reported in nine females with developmental and epileptic encephalopathy. This detailed report describes the first male with a pathogenic variant in *GLUL* presenting with developmental delays and refractory seizures.

## Introduction

More than half of individuals with a suspected Mendelian disease remain undiagnosed after analysis of the exome or genome. The incomplete diagnostic rate can be attributed to several factors, including challenges in interpreting variants for known, incompletely characterized, and novel Mendelian diseases, resulting in variants of uncertain significance, as well as technical limitations to detecting all possible variants using current sequencing technology.^1^ Novel genotype-phenotype associations are continually documented, enabling overlooked causal variants in existing sequencing data to be recovered and answers returned to families.^2^^,^^3^^,^^4^^,^^5^ Sequencing data reanalysis is reported to increase diagnostic rates by ∼10% after 2 years^6^^,^^7^ and up to 25% in some studies^6^^,^^7^ and is critical to identifying causal variants. Nearly two decades ago, the gene *GLUL*, encoding glutamine synthetase—an enzyme responsible for the conversion of glutamate to glutamine, an important energy source for cellular processes during fetal development—was first reported to cause recessive congenital glutamine deficiency (GLND [MIM: 610015]). In two unrelated individuals,^8^ homozygous missense variants, c.970C>T (GenBank: NM_001033044.4; p.Arg324Cys) and c.1021C>T (GenBank: NM_001033044.4; p.Arg341Cys), were shown to result in a complete loss of glutamine synthetase activity, presenting clinically as severe brain malformation, multiorgan failure, and infantile death.^8^ A dominant disease mechanism that is characterized by *de novo* start-loss variants in *GLUL* has recently been reported to cause developmental and epileptic encephalopathy-116 (DEE116 [MIM: 620806]).^9^ In total, nine female individuals are reported, each with a heterozygous variant resulting in start loss, either by interfering with the canonical start codon (Met1) or by splice defects in the 5′ UTR leading to skipping of the canonical start codon. In both scenarios, a truncated protein is produced by reinitiation of the translation at an alternative start codon 17 amino acids downstream (Met18), removing a 5′ degron sequence critical to the regulation of glutamine synthetase degradation. The truncated glutamine synthetase retains its enzymatic activity yet fails to undergo degradation in conditions of high glutamine, resulting in the “gain-of-stabilization” disease mechanism.^9^ Distinct from the recessive form of disease, the dominant form is associated with intractable seizures, global developmental delay, and hypotonia. Moreover, all nine individuals are alive at current ages of 16 months to 16 years.^9^ Here, we report the first adult and male proband with this condition.

## Material and methods

### Genome sequencing

This male individual and his unaffected parents were sequenced as part of the Rare Genomes Project (RGP) at the Broad Institute Center for Mendelian Genomics.^10^^,^^11^ Briefly, genome sequencing (GS) was performed on DNA purified from blood by the Broad Institute Genomics Platform on an Illumina sequencer HiSeq2500 to an average depth of 30×. Sequence reads were reassembled against the GRCh38 reference genome, and single-nucleotide variants (SNVs) and small insertions or deletions (indels) were called with the GATK Best Practice workflow (v.4.0).^12^ Structural variants (SVs) were called by the GATK-SV pipeline (v.0.28, https://github.com/broadinstitute/gatk-sv).^13^ Genomic data were analyzed by experienced RGP variant analysts in the open-source, web-based genomic analysis tool *seqr* (v.1.0-ab24c2bc).^14^ Predefined family-based searches for “*de novo*/dominant” and “recessive” variants with both “restrictive” and “permissive” thresholds for potential pathogenicity were applied as previously described.^14^ High-quality, rare (<1% allele frequency in the reference population database gnomAD), potentially damaging variants with moderate-high functional impact (loss of function, missense, and predicted splice impacting) were carefully reviewed. For variants in Mendelian disease-associated genes, the proband’s phenotype and inheritance of the variant were assessed for consistency with reported cases. External databases were cross-referenced to collect additional evidence supporting causality (e.g., OMIM, Decipher, PubMed).^15^^,^^16^^,^^17^

### RNA-sequencing

RNA from whole blood was processed using a stranded, poly(A)-tailed kit (Illumina) before being multiplexed and then underwent 150 bp paired-end sequencing, with approximately 30–50 million reads generated per sample. The sequencing data were processed with a pipeline adapted from one developed by the GTEx Consortium.^18^ Briefly, FASTQ files were aligned to the GRCh38 reference sequence using STAR-2.6.1b in two-pass mode, and duplicates were marked with Picard. RNA sequencing (RNA-seq) data were de-multiplexed and each sample sequence data aggregated into a single Picard BAM file. We quantified gene expression using RNA-Seq by Expectation Maximization (RSEM) to generate transcripts per million (TPM) values for expressed genes in each sample. Post-sequencing quality control was performed using RNA-seQC2^19^ and summarized using MultiQC.^20^ The gene counts (by TPM) were input to OUTRIDER (outlier in RNA-seq finder)^21^ and aligned BAM files input to FRASER (v.1.99)^22^ with 100 whole-blood RNA-seq control samples from the GTEx Consortium.^18^ Volcano plots were output by OUTRIDER and FRASER. For FRASER, at the filterExpressionAndVariability step, the minimum read count in at least one sample was set at 2, and the minimum ΔѰ was set to 0.05.

### Ethical considerations

All participants were consented to the Broad Institute RGP, which includes the use and sharing of data for research purposes (Mass General Brigham IRB protocol 2016P001422).

## Results

### Clinical analysis

This previously undiagnosed 25-year-old male enrolled in RGP at age 18 years with intractable seizures and global developmental delays. He was born at full term after an unremarkable pregnancy and has no family history of seizures, developmental delays, autism, or learning disabilities among his parents and two unaffected siblings (now aged 28 and 31 years).

He first presented at 5 months of age with developmental delays and failure to make eye contact or visually track objects and was diagnosed with cortical visual impairment. In infancy, he developed occasional episodes of inconsolable distress lasting 10 min with no identifiable trigger.

Seizures were first reported between ages 1 and 2 years. Myoclonic seizures predominated in early childhood (sudden jerking movements of the legs) occurring as frequently as 200–300 times a day. He also experienced primary tonic seizures (arm extension and eye deviation) and generalized tonic-clonic (GTC) seizures, mostly upon waking. Levetiracetam anti-seizure therapy was started at age 2 years with no improvement. Zonisamide was started soon after and effectively suppressed the myoclonic seizures. At age 9 years, rufinamide was trialed with no improvement. The GTC seizures occurred, on average, every 4–5 days, lasting up to 4 min, with most less than 2 min. The majority were sleep related. Recovery from these seizures has consistently been characterized by a prolonged post-ictal phase during which he is unarousable for 24 h. Occasionally, his seizures have clustered (seizing again upon emergence from the post-ictal sleep). Diazepam, later replaced by clonazepam, was administered at seizure onset to effectively reduce the incidence of clustering. Throughout the course of his adolescence, various interventions, including a ketogenic diet, medical cannabis, and cannabidiol, were introduced, with no clear benefit to seizure frequency or duration of post-ictal recovery. GTC seizure frequency fluctuated throughout his adolescent years, increasing to an average of once every 2–4 days at the onset of puberty and decreasing to an average of once every 4–5 days coinciding with the introduction of brivaracetam at age 18 years. Whether this reduction in GTC seizure frequency was in response to treatment interventions or merely the effect of having completed the pubertal transition is unclear.

He is currently under regular clinical follow-up with a neurologist for global developmental delays and intractable seizures. Now age 25, while taking zonisamide, brivaracetam, and cannabidiol, his seizures continue and are predominantly myoclonic (occurring less than 10 times per week) and GTC (occurring, on average, every 4–5 days), with occasional tonic seizures. Overall, zonisamide and brivaracetam have proven most effective in reducing the frequency of the myoclonic and GTC seizures, respectively.

His brain magnetic resonance imaging (MRI) at ages 1 and 3 years was normal. His sleep electroencephalogram (EEG) in infancy (approximately 1 year of age) demonstrated rare, central parietal spike-wave discharges during stage II of sleep; at 2 years of age, generalized spike- and slow-wave discharges in addition to biparietal spike waves over the right side; and at 3 years of age, generalized 3 Hz spike- and slow-wave discharges, including some multifocal, independent spike and slow waves. Most recently, at 24 years of age, his EEG showed frequent multifocal spike-wave discharges and slow (2.5 Hz) generalized spike and slow waves amid a diffusely slow 4–5 Hz background, most consistent with an underlying epileptogenic process affecting both hemispheres. Taken together, his multiple seizure types and EEG findings are consistent with a clinical diagnosis of Lennox Gastaut syndrome. Plasma and cerebrospinal fluid glutamine, ammonia, and cerebrospinal fluid neurotransmitter levels have not been measured.

In addition to seizures and global developmental delays, he is reported to have hypotonia, diffusely reduced muscle bulk of all four limbs, and limb contractures. To increase core strength and develop balance, he participated in daily Cuevas Medek Exercise (CME) therapy in childhood and was able, at age 3 years, to sit independently and maintain his balance in a standing position with minimal support. As he entered puberty, his mobility reduced, resulting in lower limb contractures, and he subsequently lost these skills. He is currently unable to sit or stand without assistance. At age 16, he was diagnosed with neuromuscular scoliosis of the thoracolumbar region and right hip dysplasia with subluxation on X-ray. His radiographs remain stable.

With respect to feeding, he was able to nurse successfully as an infant and consumed food orally until age 11 years. As his caloric requirements increased, he had difficulty maintaining his weight and periodically became dehydrated, requiring intravenous hydration. At age 13 years, a G-tube was placed to ensure adequate nutrition and promote weight gain.

He currently lives in a group home and requires total care. He is non-verbal with limited communication skills. He is small in stature (weight: 40.8 kg, height: 152 cm), continues with G-tube feedings, and drools excessively. His parents describe that he enjoys being around people, listening to music, being outdoors, and activities that stimulate his vestibular system, including adaptive skiing, adaptive swimming, and swings.

Table 1 provides a comparison of the phenotype in our male proband compared to the nine reported female individuals.

**Table 1:** Summary of clinical features for individuals with autosomal dominant developmental and epileptic encephalopathy due to *GLUL* start-codon-disrupting variants

	Jones et al.	This report
**Individuals**		

No. of probands	9	1
Sex	female (9/9)	male
Age at last assessment	6 months–16 years	25 years

**Seizures**		

Seizures (HP: 0001250)	Y (8/8)	Y
Age at seizure onset	10 weeks–22 months	24 months
Seizure frequency	sporadic–daily	weekly
Treatment refractory	Y (6/7)	Y

**Seizure type**		

Tonic-clonic (HP: 0002069)	Y (6/8)	Y
Tonic (HP: 0032792)	Y (2/8)	Y
Clonic (HP: 0020221)	Y (2/8)	N
Myoclonic (HP: 0032794)	Y (4/8)	Y
Atonic (HP: 0010819)	Y (1/8)	N
Absence (HP: 0011147)	Y (1/8)	N
Epileptic spasm (HP: 0011097)	Y (1/8)	N

**Seizure onset**		

Generalized (HP: 0002197)	Y (7/8)	Y
Focal (HP: 0007359)	Y (4/8)	Y

**Other features**		

Global developmental delay (HP: 0001263)	Y (9/9)	Y
Non-verbal (HP: 0001344)	–	Y
Cortical visual impairment (HP: 0100704)	–	Y
Hypotonia (HP: 0001252)	Y (9/9)	Y
Limb contractures (HP: 0003121)	–	Y
Neuromuscular scoliosis (HP: 0002650)	–	Y
Feeding difficulties (HP: 0011968)	–	Y
Growth delay (HP: 0001510)	–	Y

**Investigations**		

Abnormal MRI features	Y (7/7)	N
Enlarged perivascular spaces (HP: 0012520)	Y (5/7)	N
Thinning corpus callosum (HP: 0033725)	Y (7/7)	N
Hypomyelination (HP: 0006808)	Y (7/7)	N
Normal plasma glutamine	Y (6/8)	–
Normal CSF glutamine	Y (5/7)	–

The denominator reflects the number of probands for whom data were available. “–” denotes data that were not available. Y, yes; N, no; CSF, cerebrospinal fluid.

### Genetic analysis

Previous genetic workup included an epilepsy panel of 13 genes, followed by a childhood epilepsy panel of 40 genes (including deletions/duplications) at age 13 years, both of which were negative. Further genetic testing, including exome sequencing, was denied by insurance based on his advanced age.

At age 18 years, his family enrolled in RGP. Initial analysis of trio GS (including SNVs, indels, SVs, and tandem repeat expansions) did not identify a diagnostic variant. A *de novo* splice-disrupting variant in the 5′ UTR of *GLUL* was flagged for consideration; however, at the time of the initial analysis, *GLUL* was only associated with recessive disease. Given that a second variant in *trans* was not detected to constitute a recessive diagnosis and the proband’s clinical presentation was not in keeping with the reported phenotype and severity of GLND, the analysis remained inconclusive. Data were reanalyzed annually thereafter.

**Figure 1:**
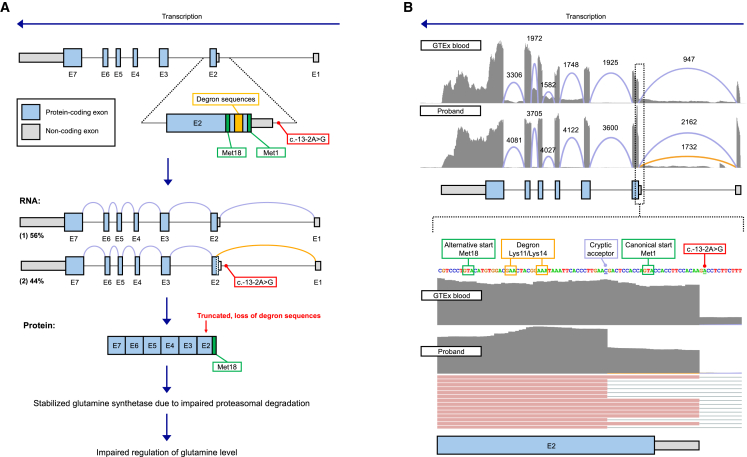
GLUL locus and the impact of the c.-13-2A>G splice-disrupting 5'UTR variant (A) Schematic of *GLUL* locus. The location of the spice-disrupting variant in *GLUL*, c.-13-2A>G (red), in the 5′ UTR non-coding region upstream of exon 2 is depicted. Two degron sequences (yellow) fall between the canonical start codon Met1 (green) and the downstream alternative in-frame start codon Met18 (green) from which translation is reinitiated. Two RNA transcript isoforms are detected in the proband, 56% with normal splice pattern (purple) and 44% with aberrant splice pattern (orange) due to the c.-13-2A>G variant. The resulting protein is truncated at the 5′ end, removing the degron sequence required to regulate glutamine synthetase degradation by the proteasome and leading to impaired regulation of glutamine level. (B) Whole-blood RNA-seq analysis. The top shows RNA-seq from normalized GTEx blood samples.^24^ The middle shows whole-blood RNA-seq from the male RGP proband. Orange line depicts the novel splice junction created by the c.-13-2A>G variant, truncating exon 2. Purple lines depict known splice junctions. The bottom shows the MANE Select transcript (GenBank: NM_001033044, GenCode: ENST00000331872) with the positions of Met1 and Met18 indicated in green, where the first 26 bases are not present in 44% of the RNA-seq reads, consistent with a heterozygous casual variant. E, exon; Met, methionine.

At age 25 years, during a reanalysis and following description of a dominant disease mechanism for *GLUL* by Jones et al., we determined the heterozygous *de novo* 5′ UTR splice-disrupting variant (c.-13-2A>G [GenBank: NM_001033044]; chr1:g.182388752T>C [GRCh38]) in *GLUL* to be causal (Figure 1A). This variant is absent from the gnomAD v.4 reference population database and was reported in one female proband (individual 8) by Jones et al. The variant is classified as likely pathogenic for autosomal dominant DEE according to the ACMG/AMP criteria,^23^ applying the following evidence codes: PS2_Moderate, PM1, PS3_Supporting, and PM2_Supporting.

### RNA-seq analysis

Annotation with the *in silico* tool SpliceAI^25^ predicts the 5′ UTR c.-13-2A>G splice variant to result in acceptor loss (delta score: 1.00, at position −2 bp) and acceptor gain (delta score: 0.97, at position −28 bp), resulting in a 26 bp deletion in the transcript. Whole-blood RNA-seq was available for the proband to confirm the splicing impact—creation of a novel splice junction between 5′ UTR exon 1 and the coding region of exon 2, resulting in the removal of the first 13 bp of the coding exonic sequence and excision of the canonical start codon at Met1 (Figure 1B). The novel splice junction is seen in 44% of the reads (1,732/2,894 exon 1-2 junctions), in keeping with the variant being heterozygous. The aberrant event was detected as a significant splice junction outlier by FRASER (Figure S1). The proband’s sample also had two significant gene expression outliers detected by OUTRIDER (*AC103810.3* and *F8A1*), though neither was considered clinically relevant to the proband’s phenotype (Figure S1).

## Discussion

We identified a *de novo* splice-disrupting variant leading to start loss in *GLUL* in the first reported male proband with DEE via a gain-of-stabilization disease mechanism recently described by Jones et al. The original study highlighted that all reported individuals were female and that the phenotype remains uncharacterized in males. The overrepresentation of females was hypothesized to be due to the reported difference in glutamine metabolism between sexes.^26^

In our analysis of undiagnosed rare disease families, we identified a male proband with a previously reported *de novo* 5′ UTR splice-disrupting variant leading to start loss in *GLUL* who exhibits phenotypic features of refractory seizures, global developmental delay, and hypotonia, as described in females in the study by Jones et al. This indicates that this genetic condition clinically affects both male and female individuals. The reason for female predominance among reported individuals remains an open question. Additionally, our male proband is non-verbal, unable to ambulate unassisted, and has cortical visual impairment, lower limb contractures, and hip dysplasia, and in contrast to the individuals reported by Jones et al., his brain MRIs in infancy and early childhood were normal. While we do not know whether the individuals described in Jones et al. shared these other characteristics, our findings suggest that both females and males with variants leading to start loss in *GLUL* have a similar phenotype.

The detection of a second proband with the c.-13-2A>G variant adds evidence in support of pathogenicity to this recurrent variant. Moreover, the identification of an additional proband with a 5′ UTR splice-disrupting variant in *GLUL* underlines the potential for the development of antisense oligonucleotides (ASOs) as therapy for allele-specific silencing of the aberrant transcript. For example, is it theoretically possible that ASO-targeted modification of the pre-mRNA mis-splicing by blocking regulatory splice elements could restore the wild-type transcript and protein function, or targeted knockdown of the mis-spliced transcript could be pursued.^27^ However, extensive experimental validation is first needed. ASOs may add to the list of plausible targeted therapies for this condition, which also includes methionine sulfoximine (MSO) therapy—an irreversible GS inhibitor trialed in rats for hyperammonemic encephalopathy that is thought to function via a similar mechanism of increased glutamine in astrocytes—as mentioned by Jones et al.

The diagnosis of this 25-year-old adult proband also highlights the importance of genetic testing across the lifespan to provide answers for affected individuals and their families, here including two older siblings, to support medical management and inform recurrence risk counseling to assist in family planning decisions.

Since we report a single male proband with this genetic condition, additional cases need to be identified to better inform the expectations of clinicians and families.

## Data and code availability

Genomic and phenotypic data from the Broad Institute of MIT and Harvard Center for Mendelian Genomics (Broad CMG) are available via the dbGaP accession number dbGaP: phs003047. Access is managed by a data access committee designated by dbGaP and is based on intended use of the requester and allowed use of the data submitter as defined by consent codes. The diagnostic *GLUL* variant had been submitted to ClinVar (accession number ClinVar: SCV005619927).

## Acknowledgments

E.C. was supported by Harvard Catalyst - The Harvard Clinical and Translational Science Center and financial contributions from Harvard University and its affiliated academic healthcare centers. S.L.S. was supported by a fellowship from the Manton Center for Orphan Disease Research at Boston Children’s Hospital. V.S.G. was supported by 10.13039/100000002NIH/National Human Genome Research Institute (10.13039/100000051NHGRI) grant K23AR083505. Sequencing and analysis were provided by the Broad CMG and funded by 10.13039/100000051NHGRI grants UM1HG008900 (with additional support from the National Eye Institute and the National Heart, Lung, and Blood Institute), U01HG011755, and R01HG009141 and, in part, by the Chan Zuckerberg Initiative Donor-Advised Fund at the Silicon Valley Community Foundation (funder https://doi.org/10.13039/100014989; grant 2019-199278) (https://doi.org/10.37921/236582yuakxy). The content is solely the responsibility of the authors and does not necessarily represent the official views of the funding agencies.

## Declaration of interests

A.O’D.L. was a paid consultant to Tome Biosciences, Ono Pharma USA, Addition Therapeutics, and Congenica and receives research funding from Pacific Biosciences. H.L.R. has received rare disease research funding from Microsoft and Illumina and compensation as a past member of the scientific advisory board of Genome Medical.

## Web resources

ClinVar, https://www.ncbi.nlm.nih.gov/clinvar/

dbGaP, https://www.ncbi.nlm.nih.gov/gap/

GenBank, https://www.ncbi.nlm.nih.gov/genbank/

Gencode, https://www.gencodegenes.org

HPO, https://hpo.jax.org

OMIM, http://www.omim.org

## Supplemental information

Document S1. Figure S1

Document S2. Article plus supplemental information
