# Supplementary material for: Male proband with intractable seizures and a de novo start-codon-disrupting variant in GLUL
Source: HGG Adv. 2025 Feb 21;6(2):100419. doi: 10.1016/j.xhgg.2025.100419 (PMC11930682; doi:10.1016/j.xhgg.2025.100419)
Supplement: Document S1. Figure S1 [file mmc1.pdf]

HGGA, Volume *6*

Supplemental information

Male proband with intractable seizures

and a *de novo* start-codon-disrupting variant in *GLUL*

Elizabeth Carbonell, Sarah L. Stenton, Vijay S. Ganesh, Jialan Ma, Grace E. VanNoy, Lynn Pais, John N. Gaitanis, Melanie C. O'Leary, Heidi L. Rehm, and Anne O'Donnell-Luria

Supplemental Material

Figure S1. RNA-sequencing analysis.

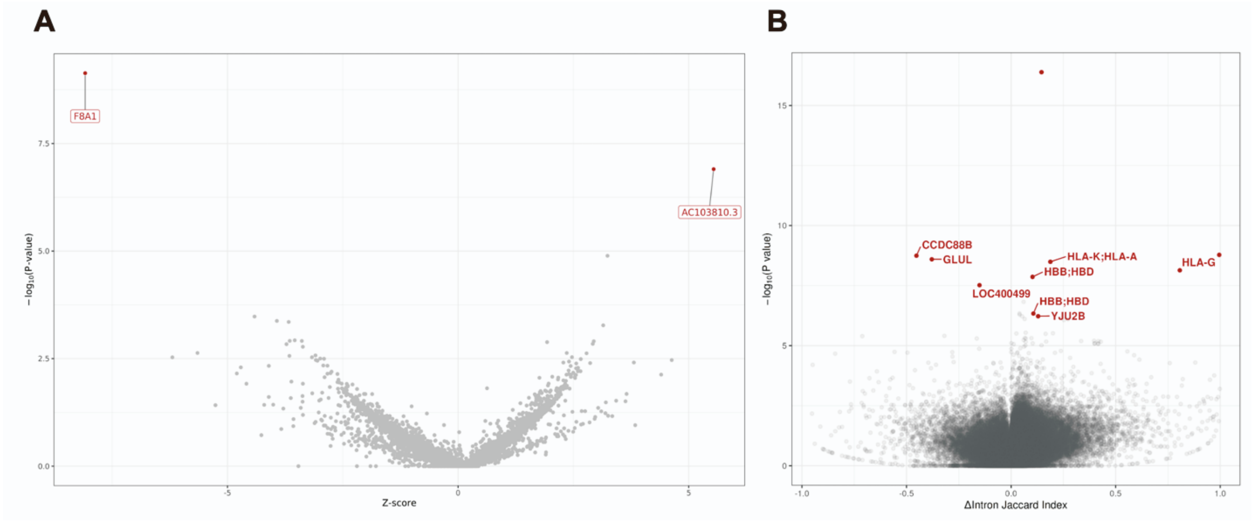

Figure S1. A. OUTRIDER volcano plot demonstrating two expression outliers. **B.** FRASER volcano plot demonstrating nine expression outliers, including *GLUL*.
